# Alveolar Ridge Preservation Using Xenograft Following Tooth Extraction: A Systematic Review and Meta-Analysis

**DOI:** 10.7759/cureus.81815

**Published:** 2025-04-07

**Authors:** Nupur Sah, Prashasti P Sarode, Ayushya Warang, Tulsi Tarase, Pandurang Gavhale, Manjiri Mirgane

**Affiliations:** 1 Periodontology, Dr. G. D. Pol Foundation, YMT Dental College and Hospital, Navi Mumbai, IND

**Keywords:** alveolar ridge preservation, bone resorption, systematic review, tooth extraction, xenograft

## Abstract

Human extraction socket healing is characterized by a series of cellular and tissue alterations; a blood clot forms inside the extraction socket quickly, which is then replaced by granulation tissue and eventually osteoid tissue. The center of the ridge shifts palatally/lingually as a result of the buccal bone plates in the maxilla and mandible. Alveolar ridge preservation (ARP) procedures were introduced to prevent alveolar ridge atrophy, preserve sufficient bone dimensions to enable implant placement in prosthetically driven positions, and maintain an acceptable ridge contour in areas of aesthetic concern. Different bone grafts have been used for ARP. Hence, this systematic review was carried out to evaluate the existing scientific literature in providing a comprehensive, quantitative analysis on the effectiveness of xenograft as a grafting material for ARP. The review adhered to the Preferred Reporting Items for Systematic Reviews and Meta-Analysis (PRISMA) guidelines 2020 and was registered in PROSPERO-CRD42024510745. Electronic databases were searched for studies evaluating the effectiveness of xenograft as a grafting material for ARP and reporting outcomes through horizontal ridge width, vertical ridge height, periodontal clinical parameters (like probing pocket depth (PPD), bleeding on probing (BOP), recession, plaque index (PI) and gingival index (GI), radiological evaluations, and associated complications. Quality assessment of included studies was evaluated through the Cochrane risk of bias (ROB)-2 tool. The standardized mean difference (SMD) was used as a summary statistic measure with a random effect model, and *p* value < 0.05 as statistically significant through Review Manager (RevMan) version 5.3 (Released 2014; The Nordic Cochrane Centre, The Cochrane Collaboration, Copenhagen). Two studies were selected for the meta-analysis, and five randomized controlled trials (RCTs) that met the eligibility requirements were included in the qualitative synthesis. There was a low to moderate ROB in the included studies. The pooled estimate showed that vertical ridge height (mid-buccal (SMD = -1.89 (-2.46-1.31), mesial (SMD = -0.18 (-0.65-0.29), and distal (SMD = -0.11 (-0.58-0.36)) decreased more with the Ext. alone group, while BOP (SMD = -0.49 (-0.96-0.01)) was more or less similar in both groups. Horizontal ridge width (SMD = 1.15 (0.97-2.05) was better preserved with ARP. Xenograft was clinically and statistically superior (p < 0.05). The funnel plot did not reveal any asymmetry, indicating the absence of publication bias in the meta-analysis. It was found that an extraction socket filled with xenograft resulted in better preservation of alveolar bone dimension, lesser ridge resorption, and provided better soft and hard tissue healing with better satisfactory results. Further clinical studies with a larger sample size and follow-up period should be carried out to assess the secondary outcomes described to obtain overall good quality evidence.

## Introduction and background

Dental extractions are necessary when a tooth cannot be adequately treated, even though maintaining the dentition should always be the preferred course of action when treating patients [1]. Loss of teeth directly affects quality of life by making it more difficult to communicate, chew food, and, in certain cases, interact with others [2].

Several animal and human clinical investigations have contributed to the present understanding of the biology behind the healing of extraction sockets [3-7]. Human extraction socket healing is characterized by a series of cellular and tissue alterations; a blood clot forms inside the extraction socket quickly, which is then replaced by granulation tissue and eventually osteoid tissue [7].

According to a systematic review, alveolar ridge width and height following extraction decreased by 3.87 mm and 1.67 mm, respectively, after three months and by 63% and 22% after six months [8]. In an animal study, it was discovered that the buccal bone ridges in the maxilla and mandible resorb considerably more than the corresponding palatal/lingual bony walls, causing the center of the ridge to shift palatally/lingually [3].

Alveolar ridge preservation (ARP) procedures were introduced to prevent alveolar ridge atrophy, preserve sufficient bone dimensions to enable implant placement in prosthetically driven positions, and maintain an acceptable ridge contour in areas of aesthetic concern [9]. Many different ARP treatment methods and graft materials have been documented during the last 25 years; however, none of these approaches are regarded as the best. Autogenous bone, allografts (demineralized freeze-dried bone allograft (DFDBA) or freeze-dried bone allograft (FDBA)), the biologics (recombinant human bone morphogenetic protein-2 (rhBMP-2) and platelet-rich plasma/platelet-rich fibrin (PRP/PRF)), xenografts (deproteinized bovine bone mineral (DBBM), porcine bone, and coralline calcium carbonate), absorbable collagen sponges, and tooth-derived grafts are among the biomaterials that have been used [1]. Additionally, studies have been conducted on guided bone regeneration (GBR) with and without bone grafts. The quality of the regenerated tissue in the socket varied significantly, despite the fact that some of the previously mentioned bone replacements were able to restrict the resorption of the post-extraction alveolar ridge to some degree. Preclinical findings indicated that the remnants of the graft frequently obstructed the natural healing process [9].

According to a systematic review, ridge preservation (RP) is useful in minimizing both horizontal and vertical shrinkage, as compared to untreated sockets. But in most of the clinical comparisons, alloplasts, xenografts, and allografts combined with alloplast (AG + AP) consistently gave good results [1].

A xenograft, also known as a heterograft, is a graft made from a different species, such as coral, equine, or bovine [10]. Natural coral and cow bone are the two sources of xenografts employed as bone replacement grafts in periodontics. Both sources resemble human bone physically and are biocompatible. The xenograft known as bovine-derived xenograft (BDX) is made up of deproteinized, sterilized bovine bone that has between 75% and 80% porosity and crystals that are about 10 mm in size and resemble cortical granules. It is believed that BDX and human bone are identical. Advantages of BDX over freeze-dried demineralized bone include the following: no donor site is needed; limitless supplies of the material are available; the material is easily handled and used in the same way as freeze-dried demineralized bone; and predictable outcomes when appropriate surgical techniques are followed, a sterile environment is maintained, and tissue is handled appropriately [1]. The inorganic porcine-derived bone xenograft exhibits strong osteoconductive activity, is a natural reproduction of autologous bone, and preserves the same intimate features (matrix and porous shape). It is both bioavailable and biocompatible. The exoskeleton is the source of natural coral graft substitutes, such as coralline calcium carbonate. The primary benefits of xenografts are their osteoconductive properties and their accessibility [11].

## Review

Protocol and registration

This review was conducted and performed according to the Preferred Reporting Items for Systematic Reviews and Meta-Analyses (PRISMA) statement [12] and registered in the Prospective Registration of Systematic Reviews (PROSPERO; registration number CRD42024510745).

Focused question

The following focused research question in the participants (P), intervention (I), comparison (C), and outcome (O) format was proposed “Does the use of xenograft show improved results in ARP when compared to spontaneous healing following tooth extraction?” Using the PICO format, the following was the focus: P (tooth extraction), I (xenografts), C (spontaneous healing), O (ARP).

Eligibility criteria

The inclusion criteria for this review encompass randomized controlled trials (RCTs) that evaluate the efficacy of ARP using bovine-derived or cortico-cancellous bone grafts in conjunction with a barrier membrane following tooth extraction. Eligible studies must focus on ARP performed before implant placement and report outcomes related to horizontal ridge width, vertical ridge height, periodontal clinical parameters (such as probing pocket depth (PPD), bleeding on probing (BOP), recession, plaque index (PI), and gingival index (GI)), radiological evaluations, and associated complications. Only studies with a minimum follow-up period of six months, involving human subjects, and providing sufficient data on the efficacy of xenografts for ARP are considered. Furthermore, the studies must be published between January 2000 and December 2023 in English language, open-access journals, and report study outcomes in terms of mean and standard deviation (SD).

Studies are excluded if they are case reports, case series, or unpublished research. Additionally, any studies conducted before 2000, published in languages other than English, or retrieved from non-open-access journals are excluded. Research involving other agents influencing outcome measures, comparisons of xenografts with other materials, animal models, in vitro studies, commentaries, or interviews is also not considered.

Information sources and search strategy

The following databases were used to conduct an electronic search until December 2023 for studies published in the last 23 years (2000-2023): MEDLINE/PubMed, Cochrane, Scopus Database, Central Register of Controlled trials (CENTRAL), EBSCO host, ScienceOpen, Google Scholar, and Open Grey databases up to and including February 2024. A manual search of the Journal of Clinical Periodontology, Journal of Periodontology, International Journal of Oral and Maxillofacial Implants, Clinical Oral Implants Research, Implant Dentistry, International Journal of Oral and Maxillofacial Surgery, Journal of Oral and Maxillofacial Surgery, Journal of Dental Research, Journal of Prosthetic Dentistry, International Journal of Prosthodontics, Journal of Oral Implantology, and International Journal of Periodontics and Restorative Dentistry was also performed. In addition to the computerized search, a manual search was conducted, and the chosen papers' reference lists were examined.

Selection strategy

A variety of combinations of Medical Subject Headings (MeSH) phrases and free text words were used in the selection process, which was based on the sorts of research AND disease AND therapy. MeSH terms adopted for electronic database search were “Longitudinal studies” OR “comparative study” OR “clinical trial” OR “controlled clinical trial” OR “randomized controlled trial,” “Tooth extraction” OR “Dental extraction” OR “Exodontia” OR “Socket preservation” OR “Socket grafting” OR “Alveolar ridge preservation” OR “Alveolar bone preservation” OR “Ridge preservation,” “Xenografts” OR “Bovine-derived bone” OR “Cortico-cancellous porcine bone” OR “Porcine bone.”

Screening process

A two-phase selection of articles was conducted. In phase one, two reviewers reviewed the titles and abstracts of all articles. In phase two, selected full articles were independently reviewed.

Quality assessment of included studies

The methodological quality among included clinical trials or RCTs was assessed using the Cochrane Collaboration risk of bias (ROB-2) tool through its various domains in Review Manager (RevMan) version 5.3 (Released 2014; The Nordic Cochrane Centre, The Cochrane Collaboration, Copenhagen) [13].

Data extraction

For all included studies, the demographic study characteristics were extracted by two independent reviewing authors through MS Excel (Microsoft Corporation, Redmond, Washington, United States) with the following headings included in the final analysis: author(s), country of study, year of study, outcome assessed, and conclusion.

Statistical analysis

Statistical analysis was conducted using Review Manager (RevMan) version 5.3, with the standardized mean difference (SMD) serving as the summary measure, and significance was determined at the threshold of p < 0.05 [14].

Assessment of heterogeneity

The Cochrane’s test for heterogeneity was employed to assess the significance of any differences in treatment effect estimations among trials. Heterogeneity was deemed statistically significant if the p-value was < 0.01 [15].

Investigation of publication bias

The study assessed publication bias using Begg’s funnel plot, which plots the effect size against the standard error. Asymmetry in the funnel plot may indicate potential publication bias [16].

Results

Study Selection

**Figure 1:**
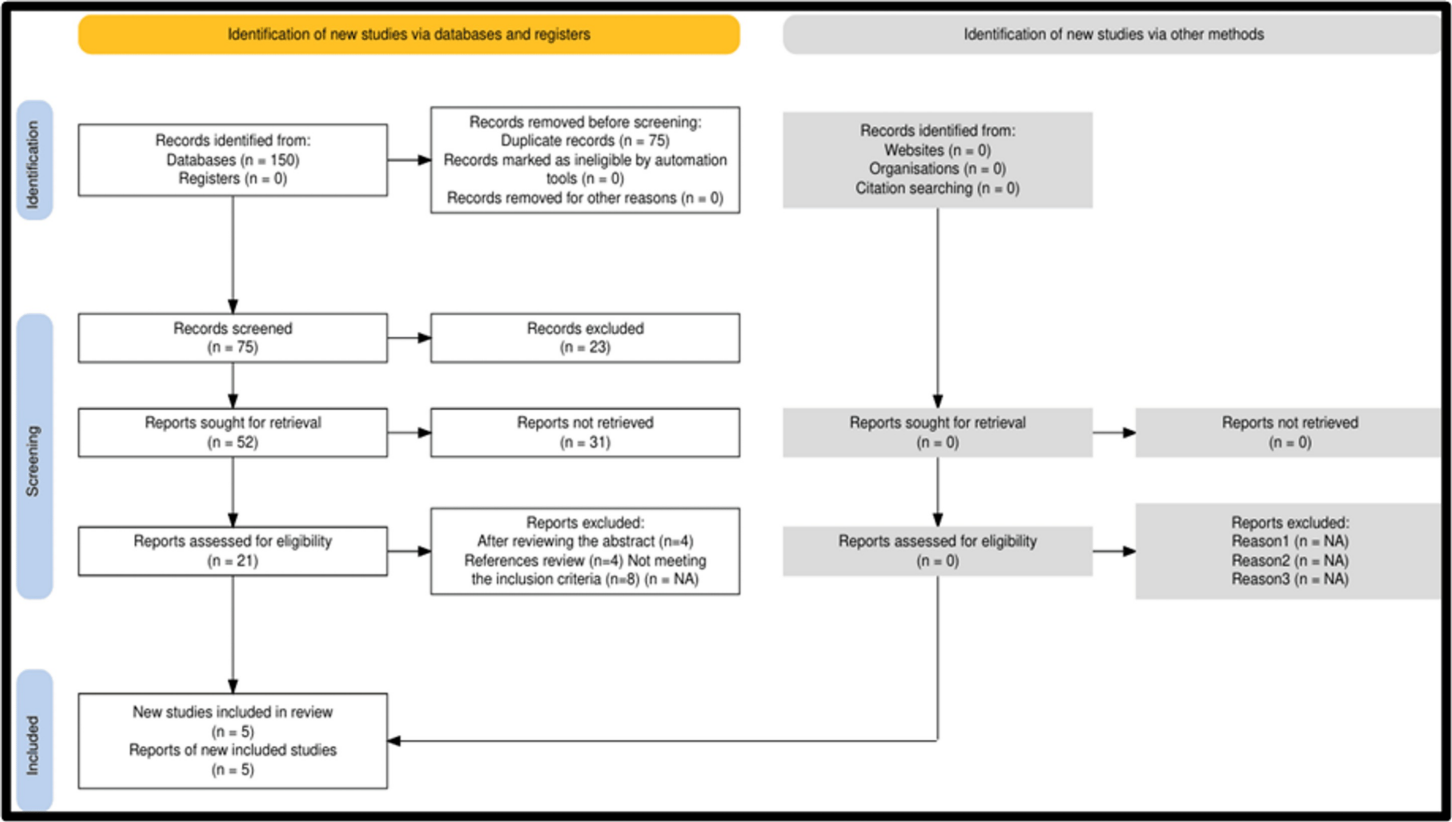
Flowchart of literature search results and study selection

Five studies were included in the review, and two studies were included in the meta-analysis, as illustrated in Figure 1.

Study Characteristics

As shown in Table 1, data were evaluated from five studies from an aggregate sample size of 200, of which 100 were allowed to undergo spontaneous healing or left undrafted, while 100 extraction sockets were filled with xenograft. All the included studies had an RCT study design (among the included studies, three studies were conducted in Italy, one study in Malaysia, and one study in Germany. Studies were assessed for a time period from the 1st month to the 36th month [17-21]. Primary outcomes were assessed in terms of horizontal ridge width, vertical ridge height, periodontal clinical parameters, and secondary outcomes in terms of radiological or histo-morphometric evaluations and associated complications as described below qualitatively.

**Table 1:** Characteristics of included studies RCT: randomized controlled trial; PPD: probing pocket depth; BOP: bleeding on probing; CBCT: cone-beam computed tomography system

Author, years of study	Country	Study design	Sample size	Intervention/comparator	Parameters assessed	Follow up (in months)	Conclusion
Barone et al., (2008) [17]	Italy	RCT	40	RP by cortico-cancellous porcine bone and collagen membrane vs extraction alone	Horizontal and vertical ridge changes, clinical parameters (PI, GI, BOP), complications	7	At the EXT sites (4.3 ± 0.8 mm), there was much more horizontal resorption than at RP sites (2.5 ± 1.2 mm). The extraction-alone group lost 3.6 ± 1.5 mm of buccal ridge height, while the ridge-preservation group lost 0.7 ± 1.4 mm. In the ridge-preserved group, the vertical change at the lingual locations was 0.4 mm, while in the extraction-only group, it was 3 mm. After seven months following tooth extraction, the histologic study revealed a considerably larger proportion of total mineralized tissue and trabecular bone in ridge-preservation sites compared to extraction-alone sites
Barone et al., (2012) [18]	Italy	RCT	40	Implant diameter and length; augmentation procedure required at implant placement for peri-implant bone defects; implant success, including implant mobility, removal of stable implants as a result of progressive bone loss and implant fracture (stability of individual implants was measured at delivery of final crown at 1, 2, and 3 years after prosthetic rehabilitation); any biologic or prosthetic complication; peri-implant marginal bone levels evaluated on intraoral, cortico-cancellous porcine bone and collagen membrane vs extraction alone		36	At the three-year follow up, both groups' cumulative implant survival rates had reached 95%. All the implants had successfully undergone osseointegration, according to radiographic assessment. During all three evaluation periods, there were no significant differences in the average loss of marginal bone between the two groups
Festa et al., (2013) [19]	Italy	RCT	30	Porcine-derived xenograft and a soft cortical membrane vs extraction alone	Clinical parameters (PPD, BOP, Rec), horizontal ridge width, and vertical height change	6	Six months later, they found that the EXT sites (3.7 ± 1.2 mm) had a significantly faster rate of reabsorption of the buccolingual/palatal dimension of the alveolar ridge than the ARP sites (1.8 ± 1.3 mm). The average decrease in vertical ridge height in the control sockets was 2.4 ± 1.6 mm at the lingual sites and 3.1 ± 1.3 mm at the buccal sites. The corresponding decreases in the test sockets were 0.5 ± 1.3 and 0.6 ± 1.4 mm. Therefore, unlike EXT, the resorption of hard tissue following tooth extraction can be decreased by using a porcine xenograft with a membrane in an extraction socket
Al Qabbani et al., (2017) [20]	Malayasia	RCT	20	Lyophilized bovine bone xenograft granules and resorbable pericardium membrane vs ext. alone	Complications (pain, paresthesia, swelling, wound still open, granulation tissue, infection). Radiological evaluation using CBCT horizontal and vertical ridge dimensions	9	The control group showed considerably higher bone resorption (1.84 mm; P < 0.05) at 9 months than the other groups (1.49 mm) at 3 months, according to investigations done across the groups. Filling extraction sockets with lyophilized demineralized bovine bone granules is essential for maintaining alveolar bone dimension because of its remarkable soft and hard tissue healing properties
Fischer et al., (2018) [21]	Germany	RCT	70	Deproteinized bovine bone mineral (DBBM) with soft collagen membrane vs extraction alone	3D volumetric changes	6	Between the two groups (deproteinized bovine bone mineral with collagen membrane, 1.26 ± 0.942, and the extraction-alone group 2.15 ± 1.349), the extraction-alone group had the greatest variation and double the amount of change in buccal contour

Qualitative assessment and analysis

Three studies assessed and evaluated the effect of graft on bone augmentation, radiological assessment of alveolar bone preservation [18,20,21]. A study investigated and compared the success rate and marginal bone loss for implants placed at grafted sites with naturally healed sites, as well as the need for additional augmentation procedures during implant placement. A control group received extraction without any grafting, while 40 patients with more than one hopeless tooth were randomly assigned to a test group that received extraction and grafting of cortico-cancellous porcine bone and were monitored for three years. From the results of the study, it was found that implants placed into grafted extraction sockets exhibited the same clinical outcome as implants placed into nongrafted sites in terms of implant survival and marginal bone loss. However, grafted sites allowed placement of larger implants and required fewer augmentation procedures at implant placement when compared to naturally healed sites [18]. Two investigations, however, assessed the changes brought about by different RP techniques as well as the effectiveness of bovine bone granules in alveolar bone socket augmentation for RP after atraumatic tooth extraction [20-21]. In one study, the test group received lyophilized bovine bone xenograft granules to fill the empty extraction socket incision, while the control group was left untreated and allowed to heal normally. The use of lyophilized demineralized bovine bone granules in socket preservation to fill in the extraction socket appears to be vital in keeping the alveolar bone dimension, as it demonstrated outstanding soft and hard tissue healing results [20]. While in the other study, it was found that the buccal contour of the extraction-alone group (2.15 ± 1.349) changed more than that of the DBBM with collagen membrane group (1.26 ± 0.942), which showed the most fluctuation [21].

Quantitative assessment and analysis

Horizontal Ridge Width

Horizontal ridge width was assessed [17,19]. After six months of healing, the ARP sites' mean initial buccolingual/palatal width dropped to 8.0 +/- 1.1 mm from 9.8 +/- 1.2 mm. The extraction sites' initial alveolar widths ranged from 9.9 +/- 1.0 mm to 6.2 +/- 1.3 mm. From baseline to final inspection, test and control sites showed a significant decrease in horizontal breadth; however, EXT sites showed a much higher horizontal reabsorption (3.7 +/- 1.2 mm) than ARP sites (1.8 +/- 1.3 mm) [19]. The horizontal ridge width of the RP group was 10.6 +/- 1.0 mm at baseline and dropped to 8.1 +/- 1.4 mm after seven months. The extraction-alone group had higher horizontal width reabsorption than the RP group, while both groups demonstrated reduction from baseline to the final assessment [17].

Vertical Ridge Height

Vertical ridge height was assessed in relation to mod-buccal, mid-buccal, mesial, and distal socket walls [17,19]. One study found that the vertical dimension changed by 0.6 +/- 1.4 mm for mid-buccal measurements and 0.5 +/- 1.3 mm and 2.4 +/- 1.6 mm for midpalatal/lingual measurements for test and control sites. Compared to ARP sites, EXT sites exhibited significantly more vertical reabsorption of the mid-buccal and midpalatal/lingual socket walls [19]. Both the buccal and lingual sites showed statistically significant changes in the vertical ridges. The extraction-alone group's vertical size at the buccal regions was 3.6 mm, whereas the RP group's was 0.7 mm. Additionally, the RP group experienced a vertical shift at the lingual regions of 0.4 mm, while the extraction-alone group experienced a vertical change of 3 mm [17].

Periodontal Parameters

Periodontal clinical parameters like the PPD, BOP, recession, PI, and GI were evaluated [17,19]. According to one study, both groups' BOPs significantly decreased at the follow-up period as compared to the baseline assessment [17]. Furthermore, the RP group showed a substantial decrease in PI from the baseline to the seven-month follow-up, but no significant difference or reduction was seen at the follow-up period [19].

Histomorphometric evaluation

Histomorphometric evaluation was done [17,20]. For the extraction-alone group, the amount of trabecular bone was 25.7% +/- 9.5%, whereas the area's connective tissue made up 59.1% +/- 10.4%. The RP group had 35.5% +/- 10.4% trabecular bone, 36.6% +/- 12.6% connective tissue, and 29.2% +/- 10.1% remaining graft material. Compared to the extraction-alone group, the RP group had substantially greater percentages of trabecular bone [17], while the other study analyzed the horizontal and vertical ridge reduction at coronal, axial, and sagittal sections at baseline, three months, and nine months. It was found that the control group (extraction group) showed greater ridge reduction at different time intervals at three different views, while no significant bone resorption was shown by the test group [20].

Complications

The post-surgical healing phase was reported [17,20]. The post-surgical healing phase was not satisfactory for the majority of patients. The most commonly reported signs and symptoms were pain and swelling [17]. While complications like pain, swelling, paresthesia, open wound, granulation tissue, and infection were considered, it was found that at the end of follow-up, swelling was the most commonly reported symptom, followed by pain and paresthesia [20].

Quality assessment of included studies

**Figure 2:**
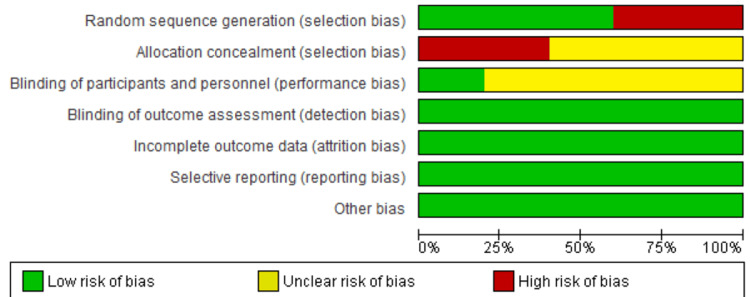
Risk of bias in individual studies

**Figure 3:**
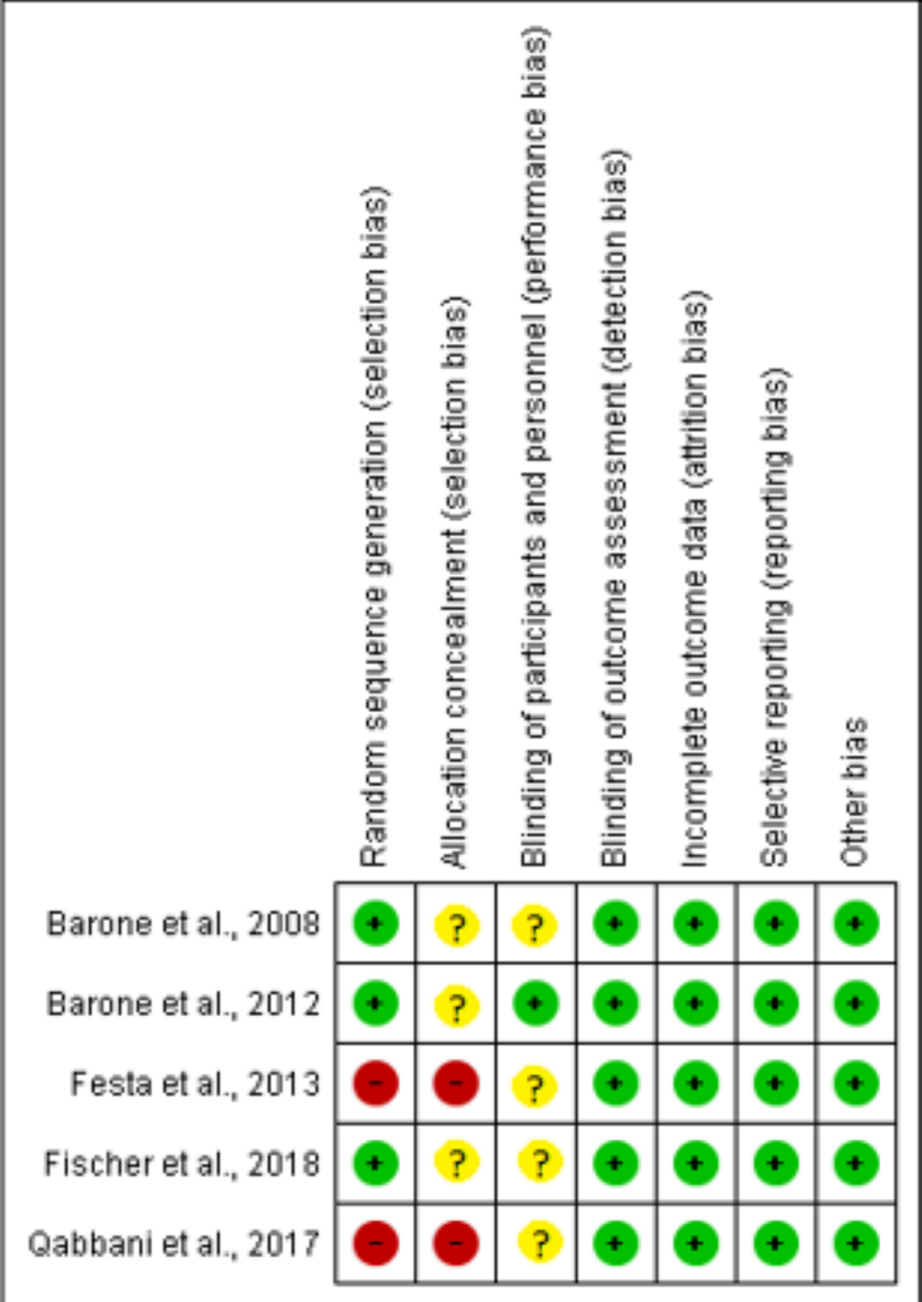
Risk of bias across studies

This assessment was conducted by using the recommended approach for assessing the RoB in studies included in Cochrane Reviews (Higgins 2011) using the tool RevMan 5.0. We used the two-part tool to address the six specific domains (namely, random sequence generation, allocation concealment, blinding, incomplete outcome data, selective reporting, and other bias). Each domain includes one or more specific entries in an RoB table. Within each entry, the first part of the tool involves describing what was reported to have happened in the study. The second part of the tool involves assigning a judgment relating to the RoB for that entry: either low risk, unclear risk, or high risk. The domains of random sequence generation, allocation concealment, blinding, incomplete outcome data, and selective reporting are addressed in the tool by a single entry for each study. We completed the RoB table for each included study in Table 2 and also depicted the same in Figures 2-3.

**Table 2:** Quality assessment of the studies included using Cochrane risk of bias tool

Sr. No.	Authors (year)	Type of study	Random sequence generation	Allocation concealment	Blinding of participants	Blinding of outcome	Incomplete outcome data	Selective reporting
1	Barone et al., (2008) [17]	RCT	Low	No information	No information	Low	Low	Low
2	Barone et al., (2012) [18]	RCT	Low	No information	Low	Low	Low	Low
3	Festa et al., (2013) [19]	RCT	High	High	No information	Low	Low	Low
4	Al Qabbani et al., (2017) [20]	RCT	High	High	No information	Low	Low	Low
5	Fischer et al., (2018) [21]	RCT	Low	No information	No information	Low	Low	Low

Meta-Analysis

Synthesis of Results

The meta-analysis was performed to assess the effectiveness of xenograft for ARP in terms of horizontal ridge width, vertical ridge height (mid-buccal, mesial, and distal), and bleeding on probing, as shown in Figures 4-13.

**Figure 4:**
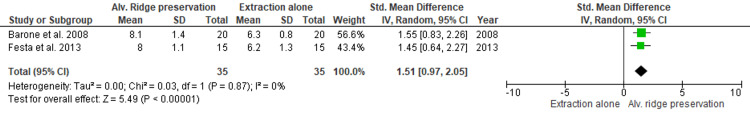
Xenograft ARP compared to extraction alone for horizontal ridge width ARP: alveolar ridge preservation

**Figure 5:**
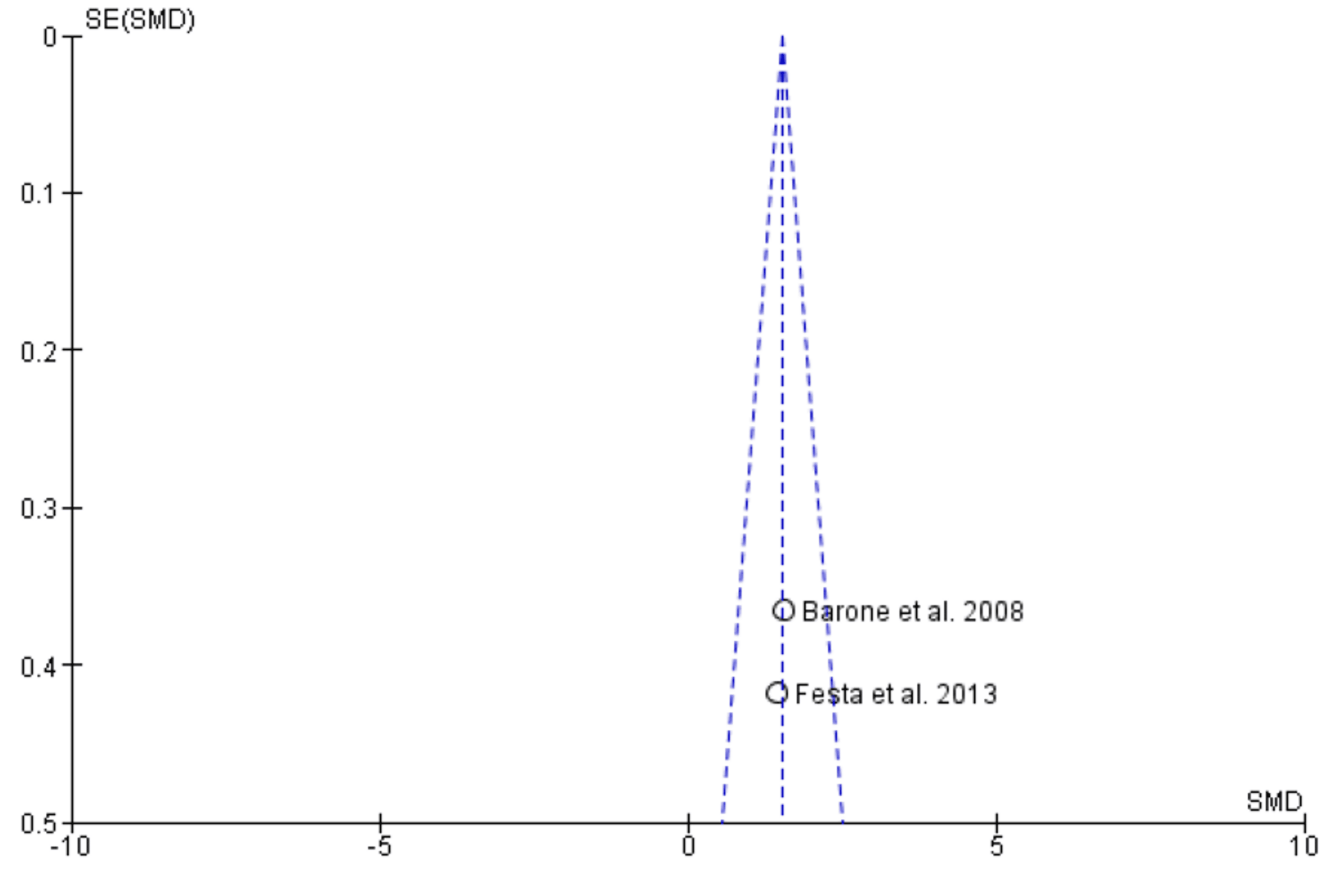
Begg’s funnel plot demonstrating the absence of publication bias

**Figure 6:**
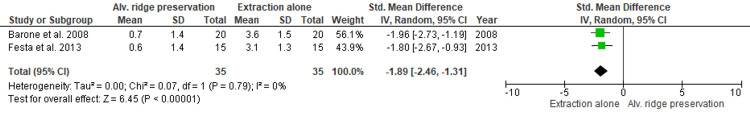
Xenograft ARP compared to extraction alone for vertical ridge height (mid-buccal socket wall) ARP: alveolar ridge preservation

**Figure 7:**
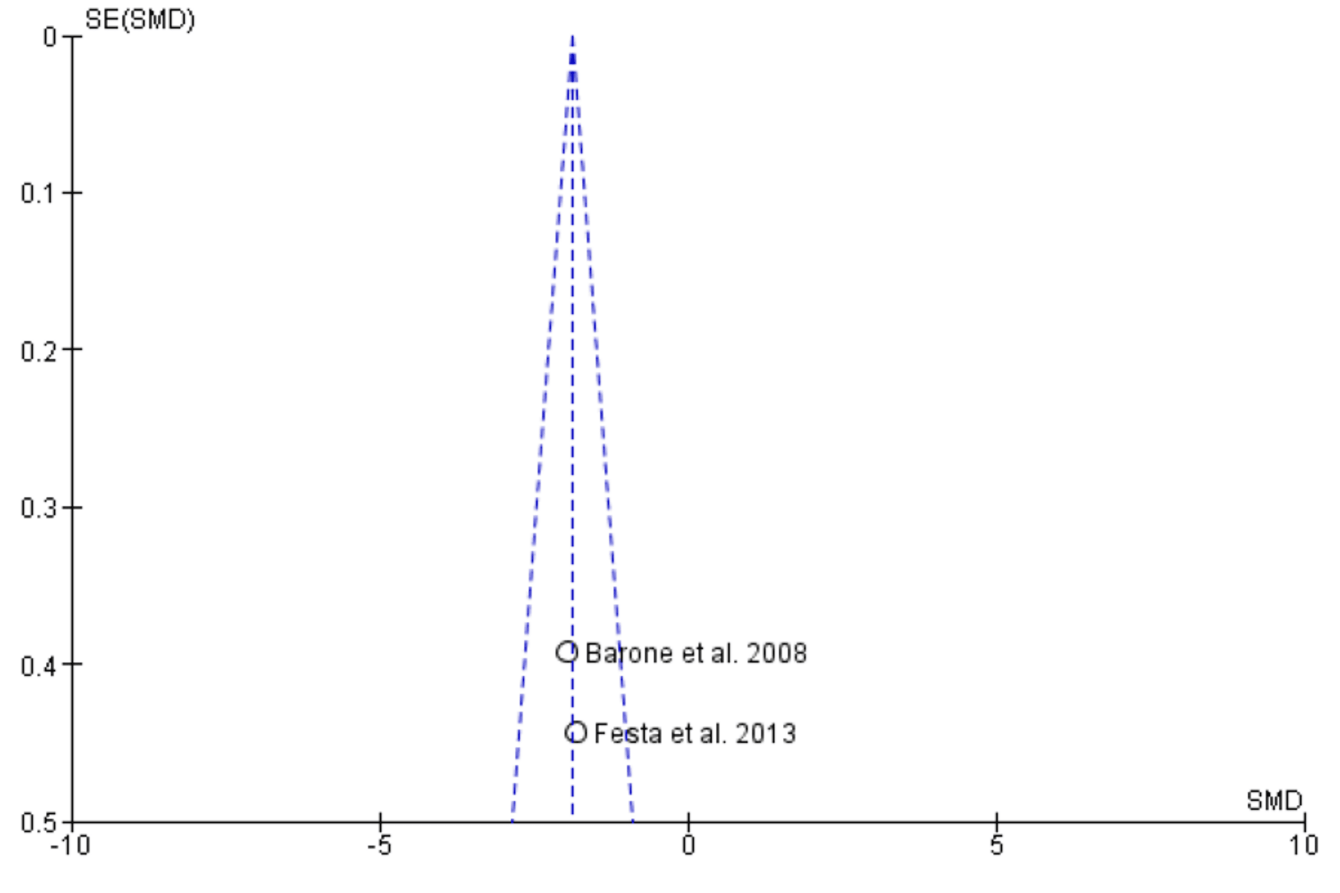
Begg’s funnel plot demonstrating absence of publication bias

**Figure 8:**
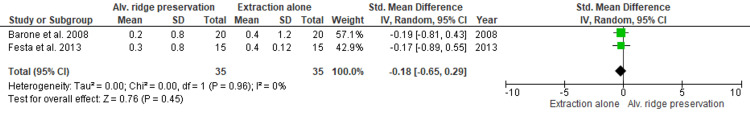
Xenograft ARP compared to extraction alone for vertical ridge height (mesial socket wall) ARP: alveolar ridge preservation

**Figure 9:**
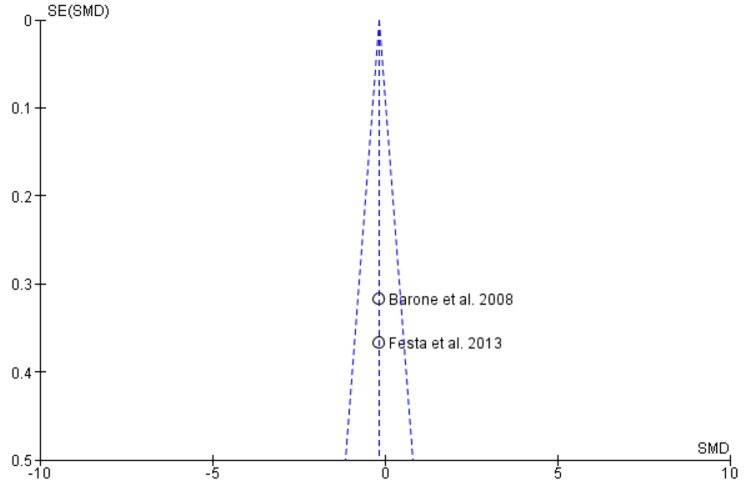
Begg’s funnel plot demonstrating absence of publication bias

**Figure 10:**
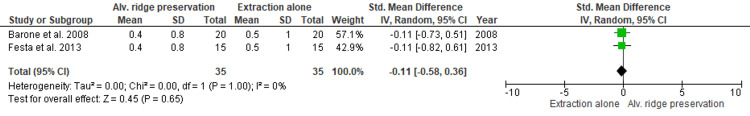
Xenograft ARP compared to extraction alone for vertical ridge height (distal socket wall) ARP: alveolar ridge preservation

**Figure 11:**
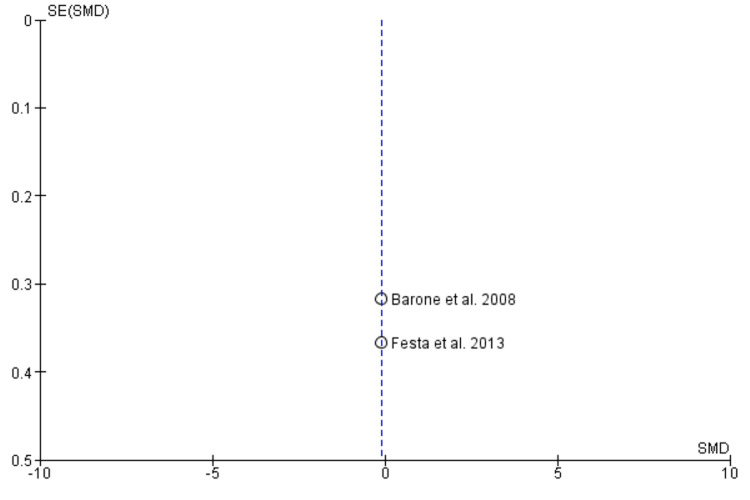
Begg’s funnel plot demonstrating absence of publication bias

**Figure 12:**
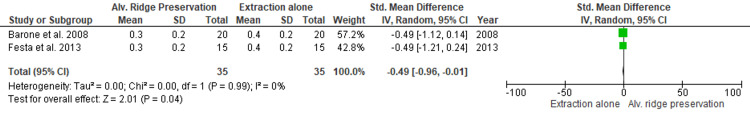
Xenograft ARP compared to extraction alone for BOP ARP: alveolar ridge preservation; BOP: bleeding on probing

**Figure 13:**
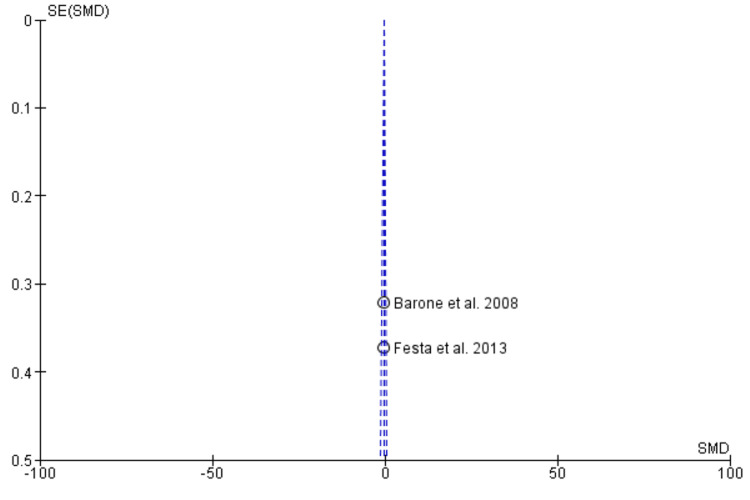
Begg’s funnel plot demonstrating absence of publication bias

Horizontal Ridge Width

Two studies containing data on 70 post-extraction sockets, of which (n = 35) sockets were evaluated by ARP by xenograft and (n = 35) sockets were allowed to heal alone for assessing effectiveness in terms of horizontal ridge width [17,19]. As shown in Figure 4, the SMD is 1.15 (0.97-2.05), and the pooled estimates favor the ARP group, signifying that the increase in horizontal ridge width on average was 1.51 times higher in the ARP group (p < 0.05). The funnel plot did not show significant asymmetry, indicating the absence of publication bias as shown in Figure 5.

Vertical Ridge Height

Mid-buccal: Two studies containing data on 70 post-extraction sockets, of which (n = 35) sockets were evaluated by ARP by xenograft and (n = 35) sockets were allowed to heal alone for assessing effectiveness in terms of vertical ridge height (mid-buccal socket wall) [17,19]. As shown in Figure 6, the SMD is -1.89 (-2.46-1.31), and the pooled estimates favor the extraction-alone group, signifying that a decrease in vertical ridge height (mid-buccal socket wall) on average was 1.89 times higher in the extraction-alone group (p < 0.05). The funnel plot did not show significant asymmetry, indicating the absence of publication bias as shown in Figure 7.

Mesial: Two studies containing data on 70 post-extraction sockets, of which (n = 35) sockets were evaluated by ARP by xenograft and (n = 35) sockets were allowed to heal alone for assessing effectiveness in terms of vertical ridge height (mesial socket wall) [17,19]. As shown in Figure 8, the SMD is -0.18 (-0.65 - 0.29), and the pooled estimates favor the heal-alone group, signifying that a decrease in vertical ridge height (mesial socket wall) on average was 0.18 times lesser in the extraction-alone group (p > 0.05). The funnel plot did not show significant asymmetry, indicating the absence of publication bias as shown in Figure 9.

Distal: Two studies containing data on 70 post-extraction sockets, of which (n = 35) sockets were evaluated by ARP by xenograft and (n = 35) sockets were allowed to heal alone for assessing effectiveness in terms of vertical ridge height (distal socket wall) [17,19]. As shown in Figure 10, the SMD is -0.11 (-0.58-0.36), and the pooled estimates favor the extraction-alone group, signifying that the decrease in vertical ridge height (distal socket wall) on average was 0.11 times less in the ARP group (p > 0.05). The funnel plot did not show significant asymmetry, indicating the absence of publication bias as shown in Figure 11.

Bleeding on Probing

Two studies containing data on 70 post-extraction sockets, of which (n = 35) sockets were evaluated by ARP by xenograft and (n = 35) sockets were allowed to heal alone for assessing effectiveness in terms of BOP [17,19]. As shown in Figure 12, the SMD is -0.49 (-0.96-0.01), and the pooled estimates favor the extraction-alone group, signifying that greater BOP on average was 0.49 times higher in the extraction-alone group (p < 0.05). The funnel plot did not show significant asymmetry, indicating the absence of publication bias, as shown in Figure 13.

Discussion

The present systematic review and meta-analysis were conducted to assess and evaluate the effectiveness of xenograft as a grafting material for ARP compared to an extraction socket left for spontaneous healing. The buccal bone generally diminishes when the associated natural tooth is lost. ARP after tooth extraction is essential to ensure adequate support for the edentulous ridge. In order to preserve or improve the original ridge dimensions and to allow an optimum implant location following tooth extraction, several materials, including autogenous, allogenous, xenogenic, and alloplastic bone substitutes and/or barrier membranes, have been used for grafting of the post-extraction socket.

A xenogenic bone substitute has also been studied that consists of cortico-cancellous porcine bone in the form of particles with a high porosity and a diameter ranging from 600 to 1,000 μm. A xenograft, also known as a heterograft, is a graft taken from another species, like coral, equines, or bovine. The most often utilized xenografts in periodontal therapy are bovine-derived bone, including anorganic bovine bone and anorganic bovine hydroxyapatite matrix (ABM) containing a synthetic cell-binding peptide. It has been stated that processing such materials removes cells, organic materials, and proteinaceous components, leaving behind inert absorbable bone scaffolding that promotes osteoblast migration, revascularization, and the production of new bone. Commercially available anorganic bovine bone (ABB) has a crystal size of about 10 nm, which is comparable to the structure of human bone. Furthermore, xenogenic bone's physical properties are equivalent to those of human cancellous bone. There are various particle sizes of bovine-derived bone, ranging from 240 to 2,000 μm. Particles between 240 and 1000 μm in size have a proportionately greater surface area, which promotes angiogenesis and osteoconduction and acts as a scaffold for the development of new bone. Studies on humans and animals have also demonstrated that ABB promotes the creation of new bone and is osteoconductive. Growth factors included in ABB may aid in the induction of new bone. However, a variety of studies on humans and animals have revealed that the resorption of ABB and its replacement with new bone seems to occur more slowly than with allografts.

The effectiveness of xenograft was assessed in terms of horizontal ridge width, vertical ridge height, periodontal clinical parameters (PPD, BOP, GI, PI, recession) as primary outcomes and secondary outcomes in terms of radiological or histomorphometric evaluations, and associated complications as patient-centered outcomes. Qualitative analysis revealed that the RP procedure resulted in better preservation of the overall alveolar wall with better soft and hard tissue healing. All the included studies demonstrated the presence of low to moderate RoB. Meta-analysis was performed on horizontal ridge width, vertical ridge height, and bleeding on probing. Through the pooled estimate, it was seen that horizonal ridge width (SMD = 1.15 (0.97-2.05) as well as vertical ridge height (mid-buccal (SMD = -1.89 (-2.46-1.31), mesial (SMD = -0.18 (-0.65-0.29), and distal (SMD = -0.11 (-0.58-0.36) was preserved better in the ARP group while BOP (SMD = -0.49 (-0.96-0.01) was seen more or less similar in both the groups. Xenograft was clinically and statistically superior (p < 0.05). The funnel plot did not reveal any asymmetry, indicating the absence of publication bias in meta-analysis. Our meta-analysis could not compare and analyze the histological, radiological, and associated complications of xenograft as a grafting material. More comparative studies should be carried out to assess these outcomes and provide an overall pooled estimate on the efficacy of xenograft as an effective graft material.

Barone et al. concluded that at the EXT sites (4.3 ± 0.8 mm), there was much more horizontal resorption than at the RP sites (2.5 ± 1.2 mm). After seven months following tooth extraction, the histologic study revealed a considerably larger proportion of total mineralized tissue and trabecular bone in the RP sites compared to the extraction-alone sites [17].

Barone et al. found that at the three-year follow-up, both groups' cumulative implant survival rates had reached 95%. All the implants had successfully undergone osseointegration, according to radiographic assessment. During all three evaluation periods, there were no significant differences in the average loss of marginal bone between the two groups [18].

Festa et al. found that six months later, the buccolingual/palatal dimension of the alveolar ridge was reabsorbed at a considerably higher rate in the EXT sites (3.7 ± 1.2 mm) than in the ARP sites (1.8 ± 1.3 mm) [19].

Studies by Al Qabbani et al. conducted across the groups revealed that the control group had significantly more bone resorption (1.84 mm; p < 0.05) at nine months than the other groups (1.49 mm) at three months. Given that it demonstrated exceptional soft and hard tissue healing, the use of lyophilized demineralized bovine bone granules in socket preservation to fill in the extraction socket is crucial in maintaining the alveolar bone dimension [20].

Fischer et al. concluded that between the two groups (DBBM with collagen membrane, 1.26 ± 0.942, and the extraction-alone group, 2.15 ± 1.349), the extraction-alone group had the greatest variation and double the amount of change in buccal contour [21].

To evaluate the efficacy of different graft materials for ARP following tooth extraction, a systematic review and meta-analysis were carried out. The following grafting materials were examined and evaluated: tooth-derived dentin, MP3Ⓡ, leukocyte- and platelet-rich fibrin (L-PRF), ApatosⓇ, Bio-OssⓇ, Bio-Oss CollⓇ, Bond-apatiteⓇ, CeraboneⓇ + PRF, FDBA, and Gen-OsⓇ. A total of 25 papers for the meta-analysis and 31 RCTs that matched the eligibility requirements were included in the qualitative synthesis. According to the review's findings, bone substitute materials can effectively lessen the alterations that occur in the alveoli following tooth extraction. It was determined, therefore, that xenograft is the finest grafting material available for preserving the alveolar ridge following tooth extraction.

The effectiveness of ARP employing xenograft and resorbable socket sealing material was assessed and evaluated through a systematic review. Up until July 2022, databases were searched, producing 10 studies for meta-analysis and 13 RCTs for qualitative analysis. Every study that was included said that there was little chance of bias. ARP via xenograft exhibited less horizontal, vertical mid-buccal, and vertical mid-lingual alveolar ridge resorption than simply spontaneous healing, according to the meta-analysis's findings. ARP combined with xenogenic bone replacement was found to be effective in lowering total volumetric alterations. 

However, there were also some limitations. Even after an unlimited search and eligibility criteria, there were very few studies with qualitative and quantitative synthesis. Only five studies were included in the final assessment. More RCTs, prospective or follow-up studies comparing ARP with xenograft are needed to evaluate the abovementioned results to show a better effectiveness between the two modalities.

## Conclusions

Thus, in the present systematic review and meta-analysis, it was found that an extraction socket filled with xenograft resulted in better preservation of alveolar bone dimension, lesser ridge resorption, and better soft and hard tissue healing with satisfactory results. Several materials, including autogenous, allogenous, xenogenic, and alloplastic bone substitutes and/or barrier membranes, have been used for grafting of the post-extraction socket to maintain the ridge dimensions. Xenografts like bovine and porcine-derived materials are also effective in preserving the alveolar bone dimension and can be used in place of other bone substitutes. Further clinical studies with a larger sample size and follow-up period are necessary to assess the secondary outcomes described to obtain overall good quality evidence. To conclude the use of xenografts for ARP appears as a promising alternative.
